# Transitional Cell Carcinoma Arising From the Renal Allograft: A Case Series and Review of the Literature

**DOI:** 10.7759/cureus.74442

**Published:** 2024-11-25

**Authors:** Taylor Laskowski, Jemma Flood, Lauren Grimm, Oyedolamu Olaitan

**Affiliations:** 1 Department of Surgery, Rush University Medical Center, Chicago, USA; 2 Department of Pediatrics, Rush University Medical Center, Chicago, USA

**Keywords:** bk virus, chronic immunosuppression, renal pathology, renal transplant surgery, urologic oncology

## Abstract

Transitional cell carcinoma (TCC) of the urinary tract appears more commonly among the transplant population. The increased incidence of TCC has been primarily associated with the male gender, BK virus (BKV), and smoking. We report a case series and comprehensive review of the literature. The comprehensive literature review was conducted via Pubmed using the keywords “transitional cell carcinoma” and “renal allograft.” At our institution, all of our cases presented with hematuria, and hydronephrosis was present in 50% (two) of our cases. BKV was detected in 75% (three) of our cases. The average time from BKV detection to TCC diagnosis was 5.6 years. The average time from transplant to TCC diagnosis was 11.25 years. Upon review of the literature, a total of 20 cases were reported of TCC arising within the renal allograft. Of those patients, 55% (11) presented with hematuria, 30% (six) had BKV, and 35% (seven) were found to have hydronephrosis. The average time from BKV detection to TCC diagnosis was 3.4 years and the average time from transplant to TCC diagnosis was 9.5 years. Given the relatively increased incidence of neoplasm among solid organ transplant recipients, painless hematuria in a renal transplant recipient should raise concern for malignancy, especially in those with prior oncogenic viral infections, history of smoking, other environmental exposures, or in those with high cumulative doses of immunosuppressive medications.

## Introduction and background

It is widely known that kidney transplantation is the optimal treatment for end-stage renal disease (ESRD), with simultaneous pancreas and kidney (SPK) transplant being an option for those with comorbid insulin-dependent diabetes. However, after solid organ transplantation, there is an increased risk of developing malignant neoplasm, including transitional cell carcinoma (TCC) of the urothelium. Often these cancers are associated with viruses, which in turn are more prevalent in immunosuppressed patients; therefore, it is postulated that immunosuppression plays a significant role in the overall risk of cancer [1].

The predisposing factors for TCC in the general population are old age, male, smoking, chronic exposure to arsenic, chemotherapy such as cyclophosphamide, radiation, aristolochic acid, Balkan nephropathy, and chronic bladder infections [2-4]. TCC of the urinary tract appears more commonly amongst the organ transplant population compared to non-transplant, with an increased incidence of 0.07%-1.9% based on various reports [2,5]. Moreover, TCC in renal transplant recipients is thought to be more aggressive, poorly differentiated, rapidly progressive, and more often fatal than in the non-transplant population [2]. Therefore, these patients often present with a higher incidence of locally advanced disease or metastatic presentation, as well as a significantly lower age at the time of diagnosis. The most common symptoms of TCC in kidney transplant recipients are painless gross hematuria and pyuria; other presentations of TCC include acute renal failure, hydronephrosis, and chronic urinary tract infection [3,4]. Cigarette smoking is the best-established risk factor for urothelial carcinoma [6]. The increased incidence of TCC has also been associated with the BK virus (BKV) [7]. BKV was first discovered by Gardner et al. in 1971 from the urine of a renal transplant recipient whose initials were B.K [3]. Initial BKV infections happen early in childhood for most people. The virus then remains latent in the kidneys throughout life and most commonly re-occurs in those receiving immunosuppression after transplantation. A majority of these tumors arise within the bladder; however, they have also been reported to appear at any location in the urinary tract, including the ureter, urethra, renal calyces, or pelvis [1]. BKV is estimated to cause a progressive kidney transplant injury in approximately 1%-10% of renal transplant patients [4]. There are many reports and reviews in the literature about bladder TCC that assess the incidence, clinical presentation, treatment, or outcomes; however, only a few cases of TCC arising from within the renal allograft have been reported [8-26]. This report highlights the unusual presentation of TCC arising from the renal allograft of kidney transplant recipients and expounds on the knowledge that has been presented in the field thus far with further discussion on etiology and the potential surveillance implications. 

Medical records of all patients diagnosed with TCC arising from the renal allograft after any kidney transplant at a single institution were reviewed up to January 2024. The initial TCC diagnosis was made through either urine cytology, contrast-enhanced computed tomography (CT) scan, magnetic resonance imaging (MRI), cystourethroscopy with retrograde pyelogram, or biopsy, with confirmatory pathology reports. BKV was diagnosed via polymerase chain reaction (PCR).

A comprehensive literature review was conducted via Pubmed using the keywords “transitional cell carcinoma” and “renal allograft.” All case reports, case series, and incidence reports of TCC arising directly from a renal allograft after any kidney transplant in adults were reviewed. Studies where individual case data could not be interpreted or where it was unclear where TCC was initially identified were excluded from the review. Reference lists of published reports were searched to find additional reports. Data on patient demographics, clinical findings, management, and outcomes were extracted. Observations are reported as frequencies, and central tendencies are expressed as mean or median with standard deviation and interquartile range, based on the distribution of data. Categorical variables are reported as number and percent frequency of occurrence. 

## Review

Case series

A total of 1,253 kidney transplants were performed at Rush University Medical Center (RUMC) between September 2012 and January 2024; 891 of those were deceased donor kidney transplants (DDKT), 242 were living donor kidney transplants (LDKT), 79 were SPK transplants, 40 were simultaneous liver and kidney (SLK) transplants and one was a simultaneous heart and kidney transplant. Of the cases presented in this article, 100% (four) were male between the ages of 44 and 65. All of our cases presented with some form of hematuria, with 75% (three) presenting with gross hematuria. Hydronephrosis was present in 50% (two) of our cases. BKV was detected in 75% (three) of our cases. The average time from BKV detection to TCC diagnosis was 5.6 years. History of tobacco use was present in 50% (two) of our cases. Only two cases had positive urine cytology for TCC. The average time from transplant to TCC diagnosis was 11.25 years. 

Case 1

A 47-year-old male nonsmoker with a past medical history of hypertension and ESRD secondary to type 1 diabetes mellitus (T1DM) who had undergone an SPK transplant. Following induction immunosuppression with solumedrol and thymoglobulin, the patient was maintained on a triple regimen of mycophenolate, tacrolimus, and sirolimus; he had no delayed function of either the kidney or the pancreas allograft. After five years, he was found to have increasing creatinine levels, raising concerns about transplant rejection. A kidney allograft biopsy at that time showed moderate interstitial fibrosis and tubular atrophy involving 40% of the cortex, attributed to polyomavirus (BK) nephropathy, focal segmental glomerulonephritis (FSGS) of the collapsing type, and chronic allograft nephropathy. He had plasmapheresis and was subsequently discharged on high-dose prednisone. Two years later, the patient was reinitiated on hemodialysis due to progressively worsening renal graft function. After over eight years post-transplant, the patient began to report episodes of gross hematuria. The patient subsequently underwent a cystoscopy, which showed normal-appearing bladder mucosa without stones, tumors, diverticula, or foreign bodies, as well as bilateral native and transplant retrograde pyelograms without any obvious abnormalities or filling defects. Urine cytology at the time, however, came back positive for high-grade urothelial carcinoma, for which the patient underwent pelvic MRI with and without intravenous (IV) contrast. A lobulated infiltrating 2.6 cm lesion concerning urothelial malignancy was found in the failed renal transplant. He subsequently underwent a nephroureterectomy with resection of the bladder cuff without complication. Pathology showed infiltrating high-grade papillary urothelial carcinoma with micropapillary and glandular features, associated with in-situ urothelial carcinoma, arising in the renal pelvis and invading the peripelvic soft tissues and renal parenchyma, along with lymphovascular invasion, confirming pT3 TCC of the renal allograft. All margins of the kidney and ureter were noted to be clear, with no lymph nodes identified. The patient remains on his initial immunosuppressive regimen as the pancreas allograft continues to function well without evidence of rejection, maintaining adequate glucose control, and insulin independence.

Case 2

A 49-year-old male nonsmoker with a past medical history of hypertension and ESRD who a underwent cadaveric renal transplant complicated by hydronephrosis from anastomotic stricture managed endoscopically. The patient was maintained on a regimen of Tacrolimus and Sirolimus with prophylactic Ganicyclovir. An ultrasound 13 years post-transplant revealed a complex cystic lesion in the upper pole of one of the native kidneys, on the ipsilateral side of the transplanted kidney. Subsequent abdominal MRI with and without contrast confirmed a 2.9 cm x 3.3 cm x 3.4 cm enhancing mass in the superior pole that was suspicious for renal cell carcinoma. He underwent open radical nephrectomy of the native kidney later that year. Pathology of the native kidney showed papillary renal cell carcinoma type 1 with resection margins free of involvement. His transplanted kidney began to progressively fail after this and 21 years post-transplant the patient began having problems with fluid overload and later nearly daily gross hematuria. MRI of the abdomen and pelvis with and without IV contrast revealed heterogeneously enhancing lesion and focal dilation of a calyx in the interpolar region of the transplanted kidney concerning urothelial carcinoma. At the time, urine cytology was negative for urothelial carcinoma. He subsequently underwent a transplant nephroureterectomy with resection of the bladder cuff and was subsequently initiated on dialysis. Pathology of the renal allograft demonstrated invasive high-grade papillary urothelial carcinoma arising in the renal pelvis invading the renal parenchyma with negative resection margins (pT3). Due to the high-grade invasive nature of his cancer, the patient was referred to the cancer center for evaluation of the potential need for systemic chemotherapy or immunotherapy. He was determined not to be a candidate for adjuvant treatment due to his comorbidities. Since then he has undergone cancer surveillance with CT chest, abdomen, and pelvis. The patient had no BKV.

Case 3

A 44-year-old male ex-smoker with a past medical history of T1DM, hypertension, and ESRD who underwent an SPK transplant. The pancreas allograft failed early from thrombosis and had an allograft pancreatectomy the following month. The renal allograft retained good function. He was on steroid-free maintenance immunosuppressants with tacrolimus and mycophenolic acid. About three years post-transplant, he was diagnosed with BK viremia via PCR, and he was started on leflunomide. During follow-up, he was found to have persistent microscopic hematuria. He underwent a cystoscopy, which was unremarkable, transplant ureteroscopy was attempted but was unsuccessful. The hematuria worsened to gross episodes, which prompted repeat cystoscopy; urine cytology from the transplanted renal pelvis washings revealed high-grade urothelial carcinoma. Ultrasound demonstrated moderate hydronephrosis of the transplanted kidney, for which a nephrostomy tube was placed. The patient subsequently underwent transplant nephroureterectomy with excision of bladder cuff and excision of nephrostomy tube tract without complication. Pathology confirmed high-grade papillary urothelial carcinoma limited to the allograft renal pelvis with vascular margins free of the tumor (pT3). He was referred to the cancer center to be evaluated for potential systemic chemotherapy or immunotherapy. He was subsequently started on an adjuvant Gemcitabine and Carboplatin chemotherapy regimen. After three cycles of adjuvant chemotherapy, it was discontinued due to intolerance. Interval abdominal/pelvic CT scan revealed no recurrence or metastatic disease. At this time, the patient moved out of state and was lost to follow-up.

Case 4

A 65-year-old male ex-smoker with a past medical history of hypertension and ESRD who had undergone DDKT. His maintenance immunosuppressants included tacrolimus and mycophenolic acid. He was diagnosed with BKV after seven years post-transplant and mycophenolic acid was switched to leflunomide. He began to have intermittent episodes of painless, gross hematuria after 15 years post-transplant. The patient underwent a cystoscopy and urine cytology which was negative. CT of the abdomen and pelvis with and without IV contrast done the following year revealed an enhancing lesion in the lower pelvicalyceal system of the transplanted kidney. Subsequent pelvic MRI with and without IV contrast confirmed an enhancing filling defect located within the lower pole collecting system of the transplanted kidney, concerning TCC. The patient was managed endoscopically with renal biopsy and fulguration without complication. Evaluation of the intraoperative specimens confirmed atypical urothelial cell pathology consistent with high-grade papillary urothelial carcinoma. Repeat ureteroscopies were performed for continued resection and surveillance of the tumor. However, the stage and depth of invasion were never able to be confirmed, the patient was unwilling to undergo transplant nephrectomy. His immunosuppressive medication was switched to Sirolimus at this time. He continued to have surveillance cystoscopy with ureteroscopy and biopsies, his pathology was intermittently suspicious for recurrence with rare clusters and scattered single atypical urothelial cells with high nuclear to cytoplasmic ratio. Repeat ureteroscopies never fully confirmed actual recurrence. At his last ureteroscopy, pathology of the aspirate of the allograft renal pelvis showed no evidence of recurrence. He was admitted to an outside hospital at the end of his 18th-year post-transplant due to dizziness and a new onset of atrial fibrillation. During the course of his hospitalization, he aspirated and went into ventricular fibrillation. Resuscitation efforts were unsuccessful. 

Review of the literature

A total of 18 articles reported one or more cases of TCC arising from the renal allograft, with a total of 20 cases in adult kidney transplant alone (KTA) and SPK recipients. Characteristics of reported and our institution’s cases are summarized in Table 1. Cases from the literature review revealed the following trends: the mean age at diagnosis of TCC was 42 years, and 14 (70%) of the cases were men. Of those patients, 15% (three) had a history of smoking. At the time of malignancy diagnosis in the renal allograft, 11 (55%) patients were experiencing hematuria, with three (27.3%) of those patients presenting with gross hematuria. Other common symptoms included flank pain (30%), nausea (10%), decrease in appetite (10%) and abdominal pain (10%). BKV was present in 30% (six) of cases during their course and the mean time from BKV diagnosis to TCC diagnosis was 3.4 years. Evidence of hydronephrosis associated with the renal allograft was found in 35% (seven) of cases. The most common immunosuppressive regimen before diagnosis of TCC was tacrolimus, mycophenolate, and prednisone (40% of cases). When specified, all cancers were staged as high-grade except for one case [11]. The most common management of TCC in these cases was transplant nephroureterectomy with resection of the bladder cuff, completed in 80% (16) of cases. Nearly all of those patients became dialysis-dependent afterward, with the rare exception of one case in which the patient immediately received another transplant. The mean time to TCC diagnosis from any renal transplant was 9.5 years. Other reported outcomes included severe allograft rejection and metastasis to muscle, lung, and colon. Of all the reported cases, 15% (three) of patients died from TCC or related complications. 

**Table 1:** Characteristics of literature review and our institution’s cases TCC: transitional cell carcinoma; BKV: BK virus

Paper	Case number	Sex	Age (yrs)	Hematuria	Hydronephrosis	Other symptoms	BKV	History of smoking	Time from BKV diagnosis to TCC diagnosis (yrs)	Time from transplant to TCC diagnosis (yrs)	Grade	Staging	Management of TCC	Death from TCC or TCC-related complications	All-cause mortality
Hevia et al. (2013) [8]	1	female	61	no	yes	fever and flank pain	no	no	n/a	12	High	pT3G4	nephroureterectomy	no	no
Hevia et al. (2013) [8]	2	female	59	no	yes	asymptomatic	no	no	n/a	1	High	pT3(m)G3	transplantectomy including ureter and bladder cuff	no	no
Saquib et al. (2009) [9]	1	male	57	yes	yes	not specified	yes	no	4	6	High	not specified	kidney transplant nephrectomy	no	no
Cox et al. (2011) [10]	1	male	58	gross	no	not specified	no	yes	n/a	4.5	High	T2N3M1	Chemo/radiation therapy	yes	yes
Mokos et al. (2006) [11]	1	male	52	yes	no	not specified	yes (native)	no	3	3	Low	pTa	right-side nephroureterectomy followed by resection of the upper quarter of allograft.	no	no
Vervloessem et al. (1998) [12]	1	male	55	microscopic	yes	flank pain and a rise in blood pressure	no	no	n/a	14	High	pT3	transplantectomy	no	no
Jensen et al. (1998) [13]	1	male	22	no	no	flank pain	no	yes	n/a	26	not specified	not specified	not specified	yes	yes
Hong et al. (2017) [14]	1	male	40	gross	yes	nausea, vomiting and flank pain	no	no	n/a	6	High	cT3N2M0	Radical graft nephroureterectomy	no	no
Kojima et al. (2015) [15]	1	male	32	gross	no	not specified	no	no	n/a	10	High	T2N0M1	renal allograftectomy (after chemo)	no	no
Dao et al. (2018) [16]	1	male	39	no	no	not specified	yes	no	9	11	High	pT1b pNx	radical kidney transplantectomy	no	no
Kamei et al. (2022) [17]	1	female	39	no	no	nausea and anorexia	no	no	n/a	9	High	pT1	allograft arterial embolization (treated with pembrolizumab d/t pt refusing allograft nephroureterectomy)	no	no
Jones et al. (2023) [18]	1	male	32	yes	no	not specified	yes	no	1	14	High	pT3N2M1	transplant nephroureterectomy with pelvic lymph node dissection	no	no
Chen et al. (2021) [19]	1	female	62	no	yes	urinary tract infection and acute renal failure	no	no	n/a	34	High	not specified	transplant nephrectomy	no	no
Yu et al. (2018) [20]	1	female	53	no	no	not specified	no	yes	n/a	7	High	T3NxMx	transplant nephrectomy	no	no
Gokce et al. (2016) [21]	1	male	30	gross	yes	not specified	not available	no	n/a	7	High	T3NxMx	transplant nephrectomy	not available	not available
Hejlesen et al. (2022) [22]	1	male	40	no	no	abdominal pain and obstructive ileus	no	no	n/a	8	High	T3	transplant nephrectomy	yes	yes
Cuenca et al. (2020) [23]	1	male	61	yes	no	pyuria and flank pain	yes	no	n/a	6	not specified	not specified	radical allograft nephroureterectomy with bladder cuff excision and lymph node sampling	no	no
Farkas et al. (2019) [24]	1	male	not specified	microscopic	no	not specified	no	no	n/a	9	High	not specified	transplant nephrectomy	no	no
Salvatore et al. (2016) [25]	1	not available	not available	no	no	diarrhea and weight loss	yes	no	not available	not available	not specified	not specified	not specified	not available	not available
Singh et al. (2018) [26]	1	female	57	microscopic	no	weakness, decreased appetite and flank heaviness	yes	no	0	3	not specified	not specified	transplant nephrectomy	no	no
Our Case #1	1	male	47	gross	no	not specified	yes	no	3	8	High	pT3	nephroureterectomy with resection of bladder cuff	no	no
Our Case #2	2	male	49	gross	yes	not specified	no	no	n/a	13	High	pT3	nephroureterectomy with resection of the bladder cuff	no	no
Our Case #3	3	male	44	microscopic	yes	abdominal pain	yes	yes	6	9	High	pT3	transplant nephroureterectomy with excision of bladder cuff	no	no
Our Case #4	4	male	65	gross	no	not specified	yes	yes	8	15	High	not specified	renal biopsy and fulguration with repeat ureteroscopies	no	yes

Discussion

TCC accounts for ~95% of bladder cancers and is the fourth most common cancer among men and the 10th most common in women in the US [27-29]. However, even in non-transplant patients, primary tumors of the renal pelvis and collecting system are rare with bladder carcinomas being 50 times more common, thought to be due to the larger surface area of the bladder mucosa [30]. The incidence of TCC after a kidney transplant has been estimated to be about three times higher than the general population, with an increased incidence of upper urinary tract origin [5]. TCC in kidney transplant patients presents a higher incidence of locally advanced disease or metastasis and lower age at diagnosis than the general population [10]. These tumors are usually diagnosed in the native bladder, native ureter/urethra, or native kidneys. In men, the most frequent location is the native kidneys, while in women TCCs occur more often in the native ureter [24]. Urothelial cancer arising within the renal allograft has reportedly been a rare occurrence. As mentioned previously, the tumor stage at the time of diagnosis in the renal transplant population is frequently more locally advanced, poorly differentiated, and diagnosed at a younger age, largely thought to be due to higher levels of immunosuppression. Therefore, regular follow-up, education, and evaluation for signs or symptoms of urothelial malignancy, including hematuria, hydronephrosis, and/or obstructive uropathy, should be recommended to prevent advanced disease.

Diagnosis of upper tract urothelial lesions presents its own challenges, specifically in renal transplant patients. Obtaining biopsies from the upper tract and renal pelvis can be challenging because of the location of the transplant ureteral orifice. Additionally, cytologic examination of aspiration of upper tract urine has been shown to be accurate in only 60% of cases [31]. This is reflective of the number of positive urine cytologic samples in patients in our case series (50%). The anatomy of transplant patients can make ureteroscopy and successful biopsy even more difficult. Ureteral strictures, especially at the ureterovesical junction, are the most common urological complication after renal transplantation [32]. With advancements in surgical techniques, the use of ureteral stent placement during renal transplantation has been shown to significantly decrease early urological mechanical complications [33]. However, prolonged stent placement has also been shown to increase complications after 30 days, with UTI and stent migration being common [34]. Should TCC be diagnosed in a renal transplant recipient, treatment options do not differ extensively from the non-transplanted population. In cases such as those presented, where TCC originates or has spread to the renal graft, radical nephroureterectomy of the transplanted kidney with resection of the bladder cuff is the standard of care, followed by a return to dialysis. Certain cases can be managed conservatively with ureteroscopy with biopsy and fulguration, with consideration given to the potential difficulty and complications as mentioned previously.

Overwhelmingly, we found in our cases and in the literature that the most common presenting symptom for TCC was painless hematuria with 100% of our institution’s cases and 55% of the literature cases reporting some sort of hematuria as the primary presenting symptom. The next most common symptom reported was flank pain with 30% of cases in the literature. Another commonly reported sign before diagnosis of TCC was hydronephrosis. Half of our cases and 35% of the literature cases reported radiographic evidence of hydronephrosis prior to diagnosis of TCC. Additionally, the average time from transplant to TCC diagnosis was found to be 11.25 years in our institution’s cases and 9.5 years in the literature. A renal transplant patient ~10 years post-transplant presenting with hematuria and/ or hydronephrosis should necessitate concern for TCC and appropriate workup.

A high incidence of malignancy and rate of aggression are associated with post-solid organ transplant immunosuppressive therapy. Some immunosuppressant drugs such as cyclosporine have been associated with the promotion of cancer progression by a direct cellular effect [8]. Increased immunosuppression has also been reported as a factor related to the development of TCC in renal transplant recipients, yet it is difficult to determine the adequate dosing of these drugs to prevent organ rejection, as well as find the balance between their side effects and immunologic capacity. Some studies, however, have reported a preventative effect of mTOR inhibitors, such as Sirolimus, on the incidence of TCC among renal allograft recipients, reinforcing the importance of immunosuppressive drug regimens among this population [27]. 

Additionally, colonization with BKV has been found to increase a patient’s risk for development of urothelial cell carcinoma. BKV DNA sequences have been reported in a range of human tumors, including the brain, pancreas, rhabdomyosarcoma, liver, Kaposi's sarcoma, and urinary tract neoplasms [9]. The mechanism of malignant transformation by the polyomaviruses is thought to be due to the presence of potent transforming genes [16]. The polyomaviruses BKV, JCV, and SV40 encode 2 viral oncogenes, the large T antigen (Tag) and the small t antigen (tag). Both viral oncoproteins can transform animal or human cells [29]. In the cases reported in this article, if the patient had BK viremia, it was an average of 5.6 years before TCC was diagnosed within the renal allograft. In our literature review, that length was shorter at 3.4 years. The observed extended latency period between the discovery of BK viremia to TCC diagnosis makes it difficult to ascertain the extent of the role the BKV has in TCC development specifically among kidney transplant recipients. 

Another aspect that this article did not specifically examine was the environmental causes of TCC. A personal history of smoking has a well-established association with TCC. Former smoking history is associated with increased long-term graft and death-censored graft loss independently from delayed graft function. That association was even stronger in recipients who restarted or continued smoking after transplantation [28]. However, specifically in renal transplant recipients there is limited literature as to how much smoking plays a role in the incidence of urothelial cancer and if there is additive risk with increased usage as is suggested above. In this article, 50% of our institution's cases had a history of smoking and in the literature, it was even less at 15%. Other environmental exposures may play a role in TCC among kidney transplant patients. The incidence of TCC within renal transplant patients in Taiwan and China is relatively high and has been linked with certain cultural herbal practices [14,35]. Previous reports have implicated exposure to aristolochic acid, a compound found in wild ginger and commonly used in Chinese herbal medicine, and concurrent use of immunosuppressive agents might create an additive effect in predisposing a renal transplant patient to TCC [35,36]. This combination has also been proven to be the causative factor in the acquisition of Balkan endemic nephropathy that has been known to slowly progress into TCC [36,37]. A disease pathology that has also been linked to BKV as mentioned above. These environmental factors should be further investigated, as they may have some bearing on future surveillance practices.

Limitations of this report include its retrospective nature and small sample size. Additional reservations should be made as we were unable to identify if transplants from our institution were ultimately diagnosed with TCC at a different hospital or were lost to follow-up. Due to this fragmentation of care considerations, our institutional case series may not be complete. However, this paper to our knowledge is the largest case series looking at a variety of factors related to TCC after transplantation including: diagnosis, time from transplant and management of TCC, timing of BKV infectivity, and duration after transplant. Additionally, being able to compare and contrast our cases to those found in the literature adds to the clinical knowledge of this rare and obscure presentation after a kidney transplant.

## Conclusions

Given the relatively increased incidence of neoplasms among solid organ transplant recipients, painless gross or persistent microscopic hematuria in a renal transplant recipient should raise concern for malignancy, especially in conjunction with radiographic evidence of hydronephrosis, even a decade after the transplant. The average time from transplant to diagnosis of TCC is 9.5 to 11.25 years. Additionally, it is important to be aware of a patient’s overall risk for TCC, especially those with risk factors such as prior BK viremia, a personal history of smoking, other environmental exposures, or chronic high-dose immunosuppression. Based on our results, when BKV is detected in renal transplant patients, it precedes a TCC diagnosis by 3.4 to 5.5 years, and this period could be considered a high-risk timeframe for patients to develop TCC. Thus, it is paramount to maintain an appropriate index of suspicion for cancer workup to allow for early intervention and improved long-term outcomes.
